# Rapid size change associated with intra-island evolutionary radiation in extinct Caribbean “island-shrews”

**DOI:** 10.1186/s12862-020-01668-7

**Published:** 2020-08-18

**Authors:** Roseina Woods, Samuel T. Turvey, Selina Brace, Christopher V. McCabe, Love Dalén, Emily J. Rayfield, Mark J. F. Brown, Ian Barnes

**Affiliations:** 1 Department of Earth Sciences, Natural History Museum, London, SW7 5BD UK; 2 Department of Biological Sciences, Royal Holloway University of London, Egham, TW20 0EX UK; 3 Institute of Zoology, Zoological Society of London, Regent’s Park, London, NW1 4RY UK; 4 School of Earth Sciences, University of Bristol, Bristol, BS8 1RL UK; 5 Department of Bioinformatics and Genetics, Swedish Museum of Natural History, SE-10405 Stockholm, Sweden; 6 Centre for Palaeogenetics, Svante Arrhenius väg 20C, SE-106 91 Stockholm, Sweden

**Keywords:** Ancient DNA, Extinct, Hispaniola, Holocene, Island evolution, *Nesophontes*, Palaeogenomics

## Abstract

**Background:**

The Caribbean offers a unique opportunity to study evolutionary dynamics in insular mammals. However, the recent extinction of most Caribbean non-volant mammals has obstructed evolutionary studies, and poor DNA preservation associated with tropical environments means that very few ancient DNA sequences are available for extinct vertebrates known from the region’s Holocene subfossil record. The endemic Caribbean eulipotyphlan family Nesophontidae (“island-shrews”) became extinct ~ 500 years ago, and the taxonomic validity of many *Nesophontes* species and their wider evolutionary dynamics remain unclear. Here we use both morphometric and palaeogenomic methods to clarify the status and evolutionary history of *Nesophontes* species from Hispaniola, the second-largest Caribbean island.

**Results:**

Principal component analysis of 65 *Nesophontes* mandibles from late Quaternary fossil sites across Hispaniola identified three non-overlapping morphometric clusters, providing statistical support for the existence of three size-differentiated Hispaniolan *Nesophontes* species. We were also able to extract and sequence ancient DNA from a ~ 750-year-old specimen of *Nesophontes zamicrus*, the smallest non-volant Caribbean mammal, including a whole-mitochondrial genome and partial nuclear genes. *Nesophontes paramicrus* (39-47 g) and *N. zamicrus* (~ 10 g) diverged recently during the Middle Pleistocene (mean estimated divergence = 0.699 Ma), comparable to the youngest species splits in Eulipotyphla and other mammal groups. Pairwise genetic distance values for *N. paramicrus* and *N. zamicrus* based on mitochondrial and nuclear genes are low, but fall within the range of comparative pairwise data for extant eulipotyphlan species-pairs.

**Conclusions:**

Our combined morphometric and palaeogenomic analyses provide evidence for multiple co-occurring species and rapid body size evolution in Hispaniolan *Nesophontes*, in contrast to patterns of genetic and morphometric differentiation seen in Hispaniola’s extant non-volant land mammals. Different components of Hispaniola’s mammal fauna have therefore exhibited drastically different rates of morphological evolution. Morphological evolution in *Nesophontes* is also rapid compared to patterns across the Eulipotyphla, and our study provides an important new example of rapid body size change in a small-bodied insular vertebrate lineage. The Caribbean was a hotspot for evolutionary diversification as well as preserving ancient biodiversity, and studying the surviving representatives of its mammal fauna is insufficient to reveal the evolutionary patterns and processes that generated regional diversity.

## Background

Accurate reconstruction of past species richness in recently extinct faunas is essential, in order to understand evolutionary history and the magnitude of human impacts on biodiversity through time. It is therefore necessary to determine the extent to which variation in faunal samples from environmental archives represents species-level differentiation, as opposed to intraspecific variation within or between conspecific populations. This can require both genetic and/or morphometric approaches [1–4], as well as comparative understanding from extant taxa about levels of variation expected within related living populations [5, 6]. In particular, uncertainty about past biodiversity levels can limit understanding of the timing and dynamics of lineage diversification seen during adaptive radiations on island systems, which are widely regarded as “natural laboratories” for studying evolution [7–9]. Insular taxa frequently experience distinctive patterns of body size change [10], but the speed at which diversifying insular lineages are able to occupy novel niches through size change remains poorly understood [11]. However, islands have experienced disproportionately high levels of human-caused extinction during recent millennia, with many extinct taxa known only from morphologically incomplete and molecularly degraded specimens preserved in the recent fossil or zooarchaeological records, hindering investigation of taxonomic baselines and evolutionary patterns [12–14].

Islands have been of relatively limited value for investigating adaptive dynamics in mammals, a well-studied group that have otherwise provided extensive insights into evolutionary trends and mechanisms, because most non-volant mammal groups show limited ability to colonise island systems through overwater dispersal [13]. The insular Caribbean is an exception. This island region has acted as an important study system for investigating ecological drivers and evolutionary dynamics of morphological differentiation in novel environments for many animal groups [9, 15], and it was also colonised by several land mammal lineages, providing an important opportunity to understand mammalian evolutionary dynamics within an insular context. However, nearly all of the region’s endemic insular mammal fauna became extinct following multiple waves of human colonisation from the mid-Holocene onwards, which led to the loss of over 100 mammal species or distinct island populations [12, 14, 16]. There is continuing uncertainty over the taxonomic status of many extinct Caribbean mammals, with many recently recognised species now considered dubious or invalid [17, 18] and other unstudied populations potentially representing undescribed species [19, 20], but molecular studies have been limited by poor DNA preservation under high thermal ages represented by the Caribbean’s hot, humid tropical conditions [21–23].

**Fig. 1:**
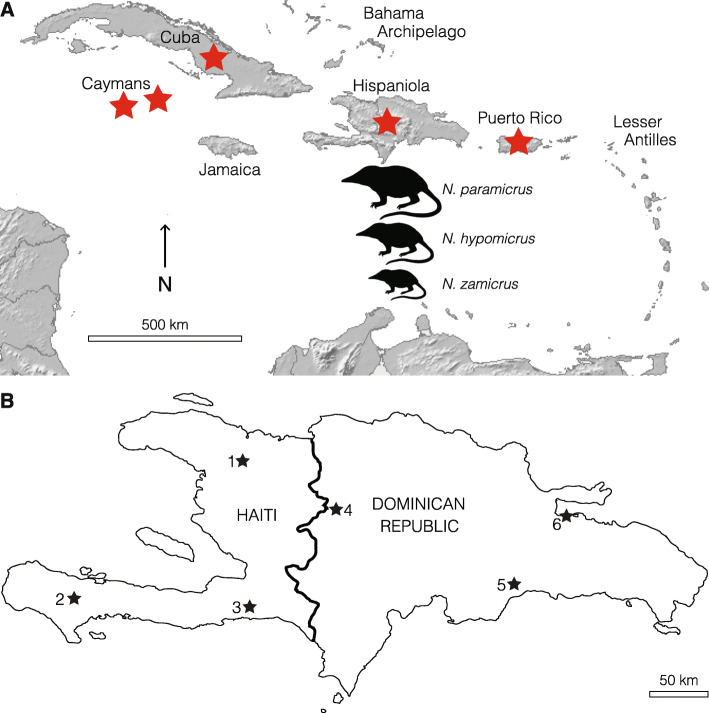
**a**, Map of the Caribbean, showing former distribution of *Nesophontes* (red stars) and relative size differences between Hispaniolan *Nesophontes* species. Basemap source: Wikimedia Commons. **b**, Map of Hispaniola, showing locations of fossil sites containing *Nesophontes* specimens included in this study. Map generated using Adobe Illustrator CS6 (www.adobe.com). Key: 1, Trou Diable; 2, Trou Fon Pelen; 3, Trou Jean Paul; 4, Cerro de San Francisco; 5, Cueva los Tres Ojos; 6, Cueva de Bosque Humido

The islands of the western Caribbean (the Greater Antilles) contain a radiation of eulipotyphlan insectivores in the endemic suborder Solenodonota, including the highly threatened solenodons (Solenodontidae) of Cuba and Hispaniola (*Atopogale cubana* and *Solenodon paradoxus*) [22, 24], and the recently extinct Caribbean “island-shrews” (Nesophontidae), which comprised the single genus *Nesophontes* (Fig. 1). Nesophontids were the smallest endemic non-volant Caribbean land mammals, and probably became extinct around 500 years ago following European-era introduction of black rats (*Rattus rattus*) [25, 26]. Some islands (Puerto Rico, Cayman Brac, Grand Cayman) contained single *Nesophontes* species, but multiple species, distinguished through morphological and/or size differences, have been described from Cuba and Hispaniola [27–30]. The validity of most Cuban species is disputed, and nesophontid diversity from this island is uncertain [31], with some authorities suggesting that only a single species was actually present [29]. Size variation shown within *Nesophontes* samples from Puerto Rico has also been interpreted as probable intraspecific variation [32, 33].

Three nesophontid species have been described from Hispaniola, the second-largest Caribbean island (divided politically into the Dominican Republic and Haiti), which are differentiated largely on the basis of size [28]: *Nesophontes paramicrus* (39-47 g), *N. hypomicrus* (21-24 g), and *N. zamicrus* (~ 10 g) (body mass estimates from ref. [14], calculated using predictive regression equations in ref. [34] based on molar measurements) (Fig. 1). All three species have been reported to co-occur in recent subfossil deposits across Hispaniola [25, 28, 35–37], suggesting they might represent a rare example of an insular mammalian adaptive radiation driven by niche availability for differing body size classes. However, the taxonomic validity and interrelationships of Hispaniolan nesophontids have not been investigated since their original description by Miller in 1929 [28], hindering understanding of past Caribbean mammalian diversity and the history and mechanism of evolutionary diversification in recently extinct endemic mammals.

Brace et al. [22] were able to recover ancient DNA (aDNA) from a *Nesophontes paramicrus* specimen from Cueva de Bosque Humido (= Cueva del Carpintero of ref. [38]; 19.077389 N, 69.477389 W), Los Haitises National Park, Hato Mayor Province, northern Dominican Republic (Fig. 1), allowing them to resolve phylogenetic relationships between the Solenodontidae and Nesophontidae. Additional *Nesophontes* specimens collected from the same rich surficial accumulation of small vertebrate skeletal elements in this cave match the morphological diagnoses of *N. hypomicrus* and *N. zamicrus* [28], with a direct date of 734 ± 24 yr BP available for a *N. hypomicrus* cranium from this accumulation [22]. In order to understand the taxonomic status, phylogenetic relationships and divergence timing of Hispaniola’s extinct eulipotyphlan fauna, we used morphometric analyses to quantify phenotypic variation in late Quaternary *Nesophontes* samples from across the island, and aDNA techniques to obtain genetic sequence data for multiple *Nesophontes* size morphs from the same late Holocene palaeontological site. Using this combined morphometric-genetic approach of across-island and within-landscape variation, we are able to reconstruct the past diversity and dynamics of evolutionary differentiation in an enigmatic group of extinct mammals, with wider implications for understanding the evolutionary processes that have generated mammalian diversity on islands.

## Results

**Fig. 2:**
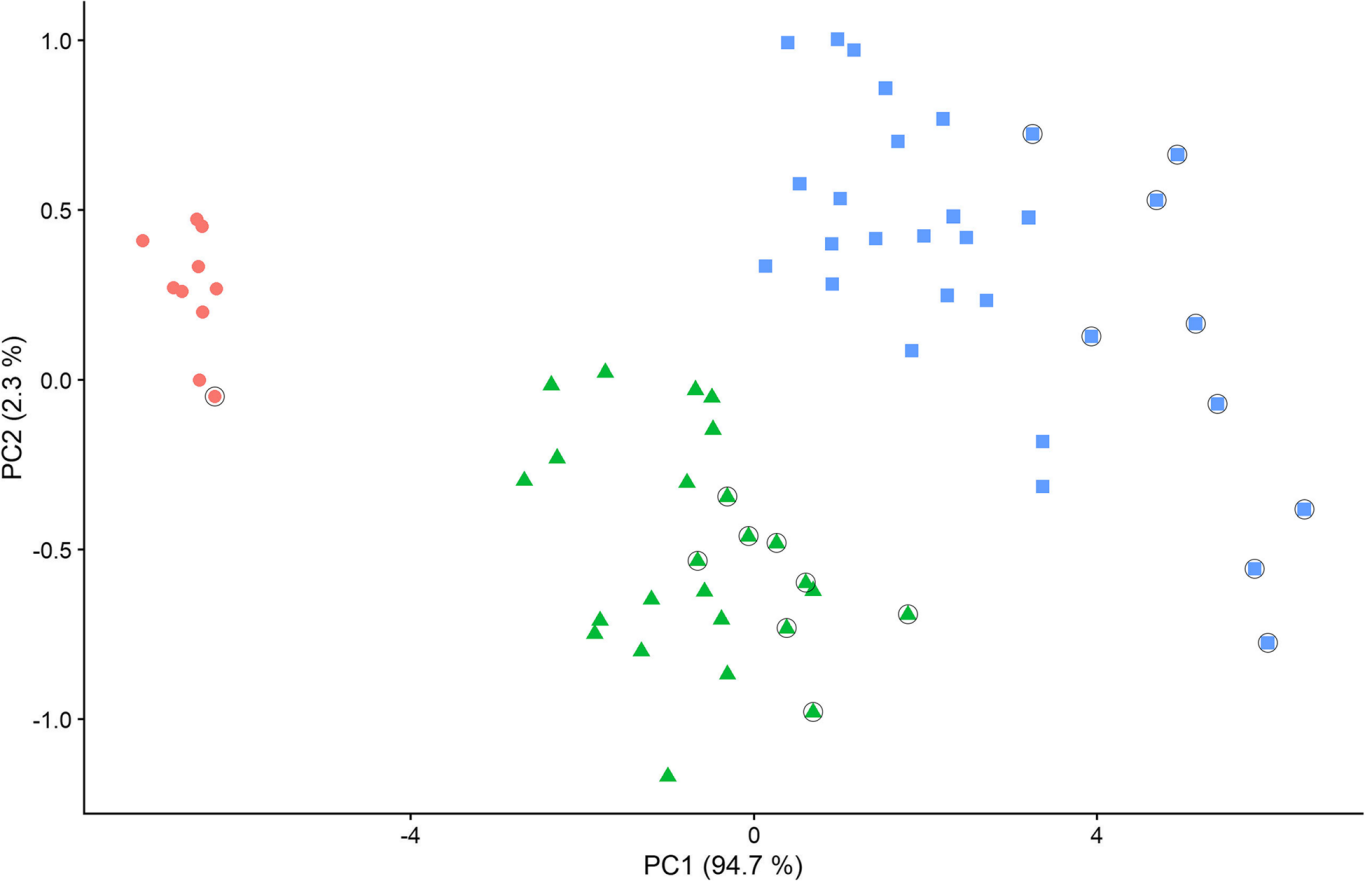
Principal Component Analysis of linear measurement data for 65 Hispaniolan *Nesophontes* mandibles, where unsupervised cluster analysis of principal component loadings identifies three discrete clusters. Specimens highlighted with open circles are from Cueva de Bosque Humido

We measured 65 *Nesophontes* mandibles from six late Quaternary fossil sites across Hispaniola, including from Cueva de Bosque Humido and other sites in both the Dominican Republic and Haiti (Fig. 1; Tables S1-S2). Cluster analysis of linear morphometric PCAs identified three clusters (parameters of automatically selected best model based on BIC = ellipsoidal, equal shape and orientation) (Fig. 2). Measured specimens from Cueva de Bosque Humido are distributed across all three clusters. Specimen classification uncertainty was < 0.05 except for one specimen (Table S2). Only the first two principal component loadings, which represent 97.0% of variation explained by PCA, were needed to achieve a stable cluster model. PC1 was highly correlated (> 0.94) with all mandibular measurements. Reanalysis using log-transformed measurements resulted in identical classification of specimens to each cluster (results not shown). There was no overlap in any measurements between clusters. ANOVAs for all measurements showed statistically significant differences between clusters, and post-hoc Tukey tests revealed statistically significant differences between all three clusters for every measurement (all *P* ≤ 0.005).

We were able to extract and sequence aDNA from two samples from Cueva de Bosque Humido that were referable on morphological criteria to the extinct endemic Hispaniolan insectivore *Nesophontes zamicrus,* although unfortunately we were unable to recover aDNA from available samples referable to *N. hypomicrus* from this site. The two *N. zamicrus* samples differed markedly in the number of reads that mapped to the *N. paramicrus* mitochondrial genome, and only the sample with the highest endogenous content was analysed further. We obtained a whole-mitochondrial genome (15,280 bp) and partial nuclear genes (APP, BMI1, CREM, PLCB4, ADORA3, APOB, ADRA2B, ADRAB2) for *N. zamicrus*.

**Fig. 3:**
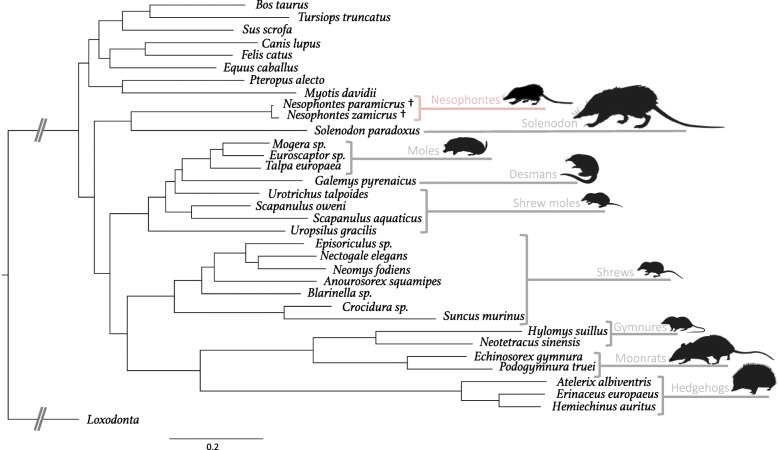
Bayesian inference phylogeny, including *Nesophontes paramicrus*, *N. zamicrus*, extant eulipotyphlan species with available whole-mitochondrial genome data and nuclear genes (APP, BMI1, CREM, PLCB4, ADORA3, APOB, ADRA2B, ADRAB2), and inclusive of all out-group taxa. Node values indicate posterior probability. Scale indicates nucleotide substitutions per site

**Fig. 4:**
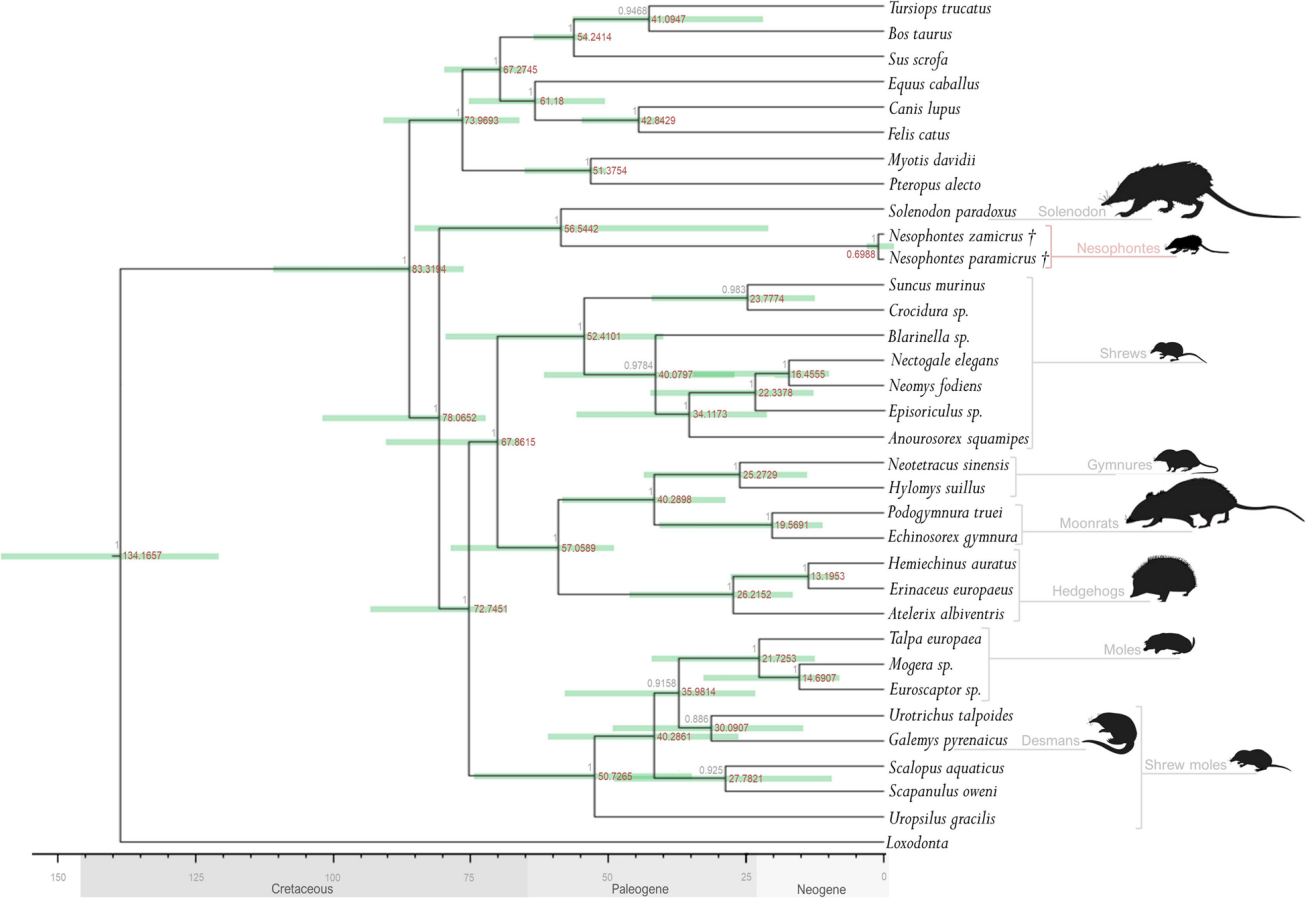
Time-calibrated phylogeny generated in BEAST using available whole-mitochondrial genome data and nuclear genes (APP, BMI1, CREM, PLCB4, ADORA3, APOB, ADRA2B, ADRAB2), showing estimated divergence dates for *Nesophontes paramicrus* and *N. zamicrus*. Estimates of median divergence dates shown above nodes in red. Node bars indicate 95% highest posterior density values. Posterior probability values shown on branches in grey

Maximum likelihood and Bayesian analyses generated almost identical well-resolved topologies, with the exception of the weakly-supported placement of the talpid genera *Galemys* and *Urotrichus* (Fig. 3, Fig. S2). Our analyses strongly support *Nesophontes paramicrus* and *N. zamicrus* as a monophyletic clade (Figs. 3 and 4, Fig. S2). These Hispaniolan *Nesophontes* species diverged from each other relatively recently, with a mean estimated divergence date of 0.699 Ma during the Middle Pleistocene (95% HPD: 0.392–1.111 Ma). Pairwise genetic distance values for *N. paramicrus* and *N. zamicrus* based on mitochondrial and nuclear genes are all relatively low (RAG1, 0.1%; BRCA1, 0.4%; BDNF, 0.6%; CREM, 2.2%; cyt *b*, 2.2%; 12S, 5.3%), but these values are all within or above the ranges of comparative pairwise genetic distances estimated between other congeneric eulipotyphlan species for which GenBank data are available (RAG1, 0.1–3.6%; BRCA1, 0.2–8.8%; BDNF, 0.0–1.5%; CREM, 0.0–0.5%; cyt *b*, 0.3–15.6%; 12S, 2.2–9.5%) (Tables S7-S12).

## Discussion

Our combined morphometric-genetic framework provides a robust new baseline for interpreting morphological variation seen in subfossil samples of nesophontid island-shrews from the large Caribbean island of Hispaniola, and our complementary analyses clarify levels of both taxonomic diversity and evolutionary differentiation within this recently extinct island mammal fauna, providing a template for future investigation of biodiversity patterns observed in environmental archives. Nesophontid mandibular samples from late Quaternary fossil sites across Hispaniola fall into three non-overlapping clusters in morphometric analysis, with between-cluster differentiation on PC1 highly correlated with our measurement indices of specimen size. Several explanations have been proposed to account for variation in within-island nesophontid samples, including sexual size dimorphism and allochronic plasticity as well as taxonomic differentiation [32, 33]. Sexual dimorphism is minimal in the extant eulipotyphlan radiation [24], within which nesophontids are phylogenetically nested [22], making this explanation unlikely, and it would not be expected to exhibit a pattern of three-way clustering in PCA. Most late Quaternary sites across Hispaniola, including several sampled for this study, have not been dated, making potential allochronic plasticity difficult to evaluate. However, the subfossil deposit at Cueva de Bosque Humido is a surficial owl accumulation with multiple direct AMS dates from within the last thousand years [22], and measured nesophontid specimens from this site fall into all three morphometric clusters identified in our PCA, showing that this pattern of morphometric differentiation was present in the late Holocene and was seen across individuals from the same landscape (Fig. 2). These results therefore provide statistical support for the former co-occurrence of three size-differentiated *Nesophontes* species on Hispaniola, as proposed in the 1920s by Miller [28] but not subsequently investigated using rigorous quantitative methods.

Complementing our morphometric findings, our genetic data indicate that two nesophontid specimens from the same late Holocene deposit in Cueva de Bosque Humido, which correspond morphologically to different described Hispaniolan *Nesophontes* species (*N. paramicrus* and *N. zamicrus*), exhibit genetic differentiation (e.g., pairwise genetic distance values for cyt *b* = 2.2%) and have a mean estimated divergence date during the Middle Pleistocene (0.699 Ma). In comparison, genetic distances for cyt *b* within extant small mammal species (including eulipotyphlans, rodents and bats), at both within-population and between-population or between-subspecies levels, are typically < 2% and usually considerably lower within a single population [39–42]. Island-endemic taxa often have even lower genetic variation compared to mainland taxa, possibly associated with stronger effects of genetic drift in small, geographically restricted island populations [43–46], making the genetic differentiation observed in our study even more noteworthy. The level of genetic differentiation seen between ~ 750 year-old nesophontid specimens from the same landscape in northern Hispaniola is thus much greater than would be expected for within-population variation in a single species, and therefore provides further evidence that these individuals likely represented different species.

Comparison of our *Nesophontes* genetic divergence data with extant eulipotyphlan insectivores is limited by the general absence of well-resolved species-level molecular phylogenies for other eulipotyphlan radiations. Pairwise genetic distances for Hispaniolan *Nesophontes* fall within the overall range of available comparative data for extant eulipotyphlan species-pairs that are diagnosed as valid species on the basis of morphological criteria. While differences between the two nesophontids are low at mitochondrial loci, pairwise genetic distances between incomplete nuclear genes analysed in our study are more comparable to other species. The youngest reported species divergences within many other eulipotyphlans are much older in comparison than observed in Hispaniolan *Nesophontes.* The youngest reported divergences date from earlier in the Pleistocene in erinaceids (*Erinaceus concolor*–*E. roumanicus*, mean = 0.89 Ma, 95% HPD = 0.4–1.4 Ma; *E. amurensis*–*E. europaeus*, mean = 1.06 Ma, 95% HPD = 0.6–1.6 Ma) [47] and in talpids (*Mogera imaizumi*–*M. tokudae*; mean = 0.8–1.0 Ma across alternative phylogenetic models; 95% HPD across all models = 0.1–2.3 Ma) [48]. Reported intraspecific divergence events for both temperate and tropical talpids also date from the Early-Middle Pleistocene (1.4 to 0.17 Ma) [42, 49]. Conversely, the youngest reported species divergences in soricids are comparable to Hispaniolan *Nesophontes*. For example, the *Anourosorex squamipes*–*A. yamashinai* divergence is dated to 0.66 Ma (95% HPD = 0.18–1.35 Ma) [50], and divergence within the *Crocidura suaveolens* complex is dated to 1.9–0.9 Ma, with different lineages exhibiting distinct morphotypes that are likely to represent separate species [51]. Similarly, divergence of 11 tropical *Sorex* species from Mexico and Guatemala is estimated to have begun 8.91 Ma, but continued until as recently as the species-level split between *S. cinereus* and *S. milleri* at 0.68 Ma [52]. These comparative data are thus consistent with recognition of species-level status for *Nesophontes paramicrus* and *N. zamicrus* based on our genetic data.

Mitochondrial sequence data for over 70 mammalian sister-species pairs have a median divergence date of 3.2 Ma under a standard (2%/MY) molecular clock. However, after using median phylogroup separation times to correct for the fact that speciation occurs over time and is not a point event, at least 50% of these mammalian sister-species speciation events are calculated to have occurred during the Pleistocene [53]. Indeed, well-recognised species pairs in many other mammal groups also show similarly young divergences to Hispaniolan *Nesophontes*, and many of these groups have much longer generation lengths than eulipotyphlans, so these temporal divergence estimates reflect an even shorter number of generations. For example, polar bears (*Ursus maritimus*) and brown bears (*U. arctos*) are estimated to have diverged 0.34–0.48 Ma [54]. Recent and rapid speciation has also been demonstrated in *Rattus*, with multiple species arising in the Middle Pleistocene [55]. Other recent species-level divergences include many boreal species such as Eurasian and American lynx (*Lynx lynx* and *L. canadensis*) and three species of lemmings (*Lemmus*) [56]. These wider comparisons with our genomic data further support recognition of species-level status for *Nesophontes paramicrus* and *N. zamicrus*.

Many of these comparative examples of other mammal species exhibiting recent divergences are from mid- or high-latitude regions, where novel environmental conditions, geologically sudden barriers to gene flow, and population movement, fragmentation and expansion/contraction associated with tracking changing habitats during Late Quaternary glacial-interglacial cycles could have promoted rapid evolutionary differentiation. In contrast, population-level divergences often date further back to the Early Pleistocene or Pliocene in many tropical environments [57]. However, Late Quaternary fluctuating climates and environmental change are known to have caused considerable disruption to Hispaniola’s ecosystems during the estimated period of divergence between *Nesophontes paramicrus* and *N. zamicrus*, including marine inundation of low-lying valleys that generated transient barriers to gene flow [58, 59], and habitat shifts between mesic and xeric conditions [60]. A shift in glacial-interglacial cyclicity around 0.8 Ma, just before the mean estimated divergence date for Hispaniolan *Nesophontes*, could have exacerbated population-level effects of these environmental changes [61]. Such geologically recent processes could potentially have driven population fragmentation and speciation in Hispaniolan *Nesophontes*.

Hispaniola’s two extant endemic non-volant land mammals, the Hispaniolan solenodon and Hispaniolan hutia (*Plagiodontia aedium*), show similar between-population pairwise genetic distances (cyt *b*: solenodon, 1.1–2.2%; hutia, 1.03–3.2%) and divergence date estimates (means = 0.436–0.594 Ma for divergences between three hutia populations) to Hispaniolan *Nesophontes* species [62, 63]. However, these taxa instead exhibit low alpha diversity and high beta (between-landscape) diversity, with separate lineages distributed allopatrically rather than sympatrically across Hispaniola’s geologically and environmentally heterogeneous landscapes. Allopatric lineages of solenodons and hutias are interpreted as representing separate subspecies rather than distinct species, because they exhibit limited (albeit statistically detectable) morphometric differentiation [18, 63], in marked contrast to the substantial body size variance seen between Hispaniolan *Nesophontes* species that co-occur in the same landscapes. Similar patterns of allopatric differentiation and high island-wide beta diversity are also seen in several other Hispaniolan vertebrate taxa, including bats [64], birds [65] and reptiles [66]. Different components of Hispaniola’s mammal fauna therefore appear to have responded in different ways to Late Quaternary environmental change, exhibiting drastically different biogeographic patterns and rates of morphological change. Studying only the surviving representatives of this remarkable insular mammal fauna is thus unable to reveal the full scope of the evolutionary patterns and processes that generated regional Caribbean diversity.

We were able to compare ancient DNA data for the largest-bodied and smallest-bodied representatives of Hispaniola’s radiation of *Nesophontes* species, and the rapid rate of morphological evolution that we demonstrate in our palaeogenomic analyses of Hispaniolan *Nesophontes* is remarkable. It remains difficult to contrast this evolutionary rate against patterns seen more widely across the Eulipotyphla, because although this is one of the most species-rich mammalian orders, with 530 currently recognised extant species [24], relatively few comparative molecular studies investigating the phylogenetic relationships across other eulipotyphlan clades are available, and relatively few of these available studies have estimated divergence times between sister species rather than between higher-order groupings [67–70]. Greater interspecific variation in body mass compared to that between Hispaniolan *Nesophontes* species is seen within several morphologically and ecologically broadly comparable genera of soricids (“true shrews”), such as *Sorex* (smallest, *S. minutissimus*, 1.4–4 g; largest, *S. bendirii*, 10–21 g) and *Suncus* (smallest, *S. malayanus*, 1.1–2.4 g; largest, *S. murinus*, 23.5–147.3 g) [24]. However, small-bodied and large-bodied species are not closely related to each other within these soricid genera, in contrast to representing rapid recent evolutionary divergence of body sizes as seen in Hispaniolan *Nesophontes* [71, 72].

The rapid morphological change observed between *N. paramicrus* and *N. zamicrus* is instead more characteristic of evolutionary rates seen in other insular taxa. Island species adapt to novel conditions found in insular environments (e.g., reduced interspecific competition, resource limitation) through changes in size and morphology that are often rapid and large-scale, driven by processes such as competitive character displacement and character release [10, 13, 73, 74]. Evolutionary rates for insular mammals are shown to be accelerated in comparison with mainland species, with faster evolution not only a response to initial colonisation but also occurring over thousands of years [11]. However, the limited number of studies of body size change in extinct island vertebrates have focused on ‘charismatic’ large-bodied species [75–77], and our study therefore provides an important new example of rapid body size change in a now-extinct small-bodied insular vertebrate lineage.

## Conclusions

Despite adverse preservational conditions for ancient biomolecules in Caribbean environments, we were able to recover aDNA successfully from the extinct Hispaniolan island-shrew *Nesophontes zamicrus*, the smallest known non-volant endemic Caribbean land mammal [14]. This is only the second extinct Caribbean eulipotyphlan species for which genomic data are available, and our data represent some of the first aDNA sequences that have been recovered for any extinct vertebrates known from the rich Holocene subfossil record of the Greater Antilles [78, 79].

Although it has been suggested that even the largest Caribbean islands only supported minimal species richness of endemic nesophontids [29], our combined morphometric and genetic analyses together demonstrate that Hispaniola supported a diverse late Quaternary nesophontid fauna comprising three co-occurring species, and that *Nesophontes paramicrus* and *N. zamicrus* diverged through rapid morphological evolution. The Late Quaternary Caribbean land mammal fauna thus consisted not only of ancient evolutionary lineages, such as the eulipotyphlan suborder Solenodonota and its two endemic families Nesophontidae and Solenodontidae [22, 80], but also recent evolutionary radiations that may have been driven by Quaternary environmental change, with Hispaniola representing a cradle of diversity as well as a museum of diversity [81]. This pattern of diversification is comparable to that seen on other ancient island terranes such as New Zealand, which also contains ancient vertebrate lineages such as kiwis (Apterygidae) that have similarly undergone recent species radiations [82, 83]. The insular Caribbean was therefore an important hotspot for the ongoing generation of new mammal species and evolutionary novelty, until the recent loss of much of this diversity following human arrival.

## Methods

### Morphometric analysis

We measured mandibles of adult *Nesophontes* specimens (defined as individuals showing complete dental eruption) in the Vertebrate Paleontology Collection, Florida Museum of Natural History (47 specimens) and the Palaeontology Collections, Natural History Museum, London (18 specimens) (Table S2). We took 13 measurements on each specimen (Table S1), using digital calipers accurate to 0.02 mm.

All statistical analyses were carried out in R v 6.3.0 [84]. We conducted PCA on our measurement dataset using the prcomp() function in R, first using raw linear data, and then using log-transformed measurements to account for the potential confounding influence of variation in size [85]. We used the ‘mclust’ package in R [86] to conduct hierarchical model-based cluster identification using principal component loadings, and determine if mandibles grouped into discrete clusters. We sequentially added principal component loadings until a distinct pattern of cluster classification was observed. Cluster models were unsupervised to ensure no bias in specimen classification, entailing no prior assignment of any specimens to a specific cluster, or constraint on the maximum number of potential clusters (to a maximum of nine by default in ‘mclust’). We then conducted ANOVAs to assess variation in each individual mandibular measurement between clusters, and conducted Tukey post-hoc tests to describe differences between clusters.

### Ancient DNA analysis

We sampled seven *Nesophontes* partial crania from Cueva de Bosque Humido, identified as *N. hypomicrus* (*n* = 2) and *N. zamicrus* (*n* = 5) based on morphometric criteria given in ref. [28], by powdering the bone using a Mikro Dismembrator. We conducted extractions and Next Generation Sequencing library builds in a dedicated aDNA laboratory at the Natural History Museum, London. Each sample was processed using utensils cleaned with bleach and UV-treated before and after use, in order to limit cross-contamination. Extraction protocol followed ref. [87] and included the use of proteinase K for bone digestion and silica-spin columns for DNA purification. Single-index double-stranded DNA libraries were built following protocols in ref. [88]. Negative extraction and library-build controls were included during each process.

*Nesophontes* samples were screened using the NHM’s Illumina NextSeq 500 to assess DNA quality. Reads were de-multiplexed by index, adapters were removed, and paired end reads were quality checked and merged using default settings on CLC genomics workbench v.8 (CLC Bio-Qiagen, Aarhus, Denmark). Reads were then mapped using CLC genomics workbench to the previously-sequenced *N. paramicrus* whole-mitochondrial genome and nuclear genes (Table 1, Fig. S1). Mapping parameters were as follows: length fraction = 0.8, similarity fraction = 0.8. The highest-quality sample (based on number of reads mapping to the *N. paramicrus* mitochondrial genome; estimated endogenous content = 0.633%) was sent to the Department of Bioinformatics and Genetics at the Swedish Museum of Natural History for deeper sequencing on an Illumina HiSeq X. We concatenated the resulting whole-mitochondrial genome and nuclear genes [89, 90], and aligned our sample with data from GenBank for 25 extant eulipotyphlan genera and 10 eutherian mammal outgroup taxa (Table S3). We applied PartitionFinder [91] to choose the most appropriate partitioning scheme and best-fit evolutionary models (Table S4).

**Table 1:** Summary statistics from CLC genomics workbench for *Nesophontes zamicrus* sample reads mapped to *N. paramicrus* mitochondrial genome

Summary statistic	Value
Total paired reads	919,533,272
Total nucleotides	53,008,411,080
Reference length	15,753
Total consensus length	15,456
GC contents (%)	37.12
Total reads mapped to reference	58,248
Mean read length	49.23
Fraction of the reference covered	0.98
Minimum coverage	0
Maximum coverage	1409
Average coverage	65.87

We constructed Bayesian trees using MrBayes [92] with four chains (three heated, one cold) that were run for 1 × 10^6^ generations, sampling every 1 × 10^3^ generations with a burn-in period of 250 trees. We generated a maximum likelihood tree with bootstrap support values using RAxML v.8 [93] implemented in CIPRES Science Gateway v.3 [94, 95]. We conducted divergence dating using fossil calibrations and priors taken from refs [96, 97] (Table S5), jointly estimating phylogeny and divergence dates under an uncorrelated relaxed lognormal clock [98]. Due to convergence issues in BEAST for large-scale genomics datasets, we employed the Hasegawa-Kishino-Yano (HKY) model of sequence evolution [99] with a gamma distribution of rates across sites. We used a Yule model of speciation; we also ran a birth-death model for comparison and generated an identical topology. Taxa sets for all monophyletic clades (including both outgroup and ingroup taxa) were constrained. We then applied lognormal priors based on available fossil data (Table S3) to seven taxa sets, with the remaining taxa sets left as the default priors. Clock rate priors were set to uninformative uniform distributions (upper = E100, lower = E12). We left all other priors as default values in BEAUti v.1.8.3 [100]. We ran analyses for 25 million generations, sampling every 1000 generations. We used Tracer v.1.6.0 (beast.community/) to assess convergence and effective sample size for all parameters after a burn-in of 10%. We generated a maximum credibility tree in TreeAnnotator v.1.8.3 [98], using trees sampled in the prior distribution.

Pairwise genetic distances between *Nesophontes* species were estimated using MEGA v.4 [101]. We used four nuclear genes (BDNF, BRCA1, CREM, RAG1) and two mitochondrial genes (cyt *b*, 12S) for genetic pairwise distance analyses (Table S3). We then determined pairwise sequence divergences (calculated as Kimura two-parameter distances) for Hispaniolan *Nesophontes* species, and also comparatively for congeneric pairs/groupings of 49 extant eulipotyphlan species currently recognised as valid [24], in 11 genera, using data from GenBank (Table S6).

## Supplementary information

**Additional file 1: Figure S1.** (a) Distribution of read lengths in paired-reads dataset. (b) Level of coverage across reference mitochondrial genome in mapped-reads dataset. **Figure S2.** Maximum likelihood phylogeny generated using RAxML, including *Nesophontes paramicrus*, *N. zamicrus*, and extant eulipotyphlans with available whole mitochondrial genome data. Node values represent bootstrap support (100 replicates). **Table S1.** Morphometric measurements taken on Hispaniolan *Nesophontes* mandibles. **Table S2.** Measurement and PCA data for Hispaniolan *Nesophontes* mandibles. **Table S3.** Genes used in phylogenetic analysis, including GenBank accession numbers. Asterisk indicates chimeric taxon made up of multiple species. **Table S4.** Evolutionary models chosen for each gene in the alignment using PartitionFinder. **Table S5.** Fossil constraints and priors used in divergence date analysis. **Table S6.** Eulipotyphlan species used in pairwise genetic distance analysis. **Table S7.** Pairwise distances for eulipotyphlan sister species pairs in the mitochondrial cyt *b* gene, showing number of base differences per site between sequences. **Table S8.** Pairwise distances for eulipotyphlan sister species pairs in the mitochondrial 12S gene, showing number of base differences per site between sequences. **Table S9.** Pairwise distances for eulipotyphlan sister species pairs in the CREM (cAMP responsive element modulator) protein-coding nuclear gene, showing number of base differences per site between sequences. **Table S10.** Pairwise distances for eulipotyphlan sister species pairs in the BRCA1 (Breast Cancer 1) protein-coding nuclear gene, showing number of base differences per site between sequences. **Table S11.** Pairwise distances for eulipotyphlan sister species pairs in the RAG1 (recombination activating gene 1) protein-coding nuclear gene, showing number of base differences per site between sequences. **Table S12.** Pairwise distances for eulipotyphlan sister species pairs in the BDNF (brain derived neurotrophic factor) protein-coding nuclear gene, showing number of base differences per site between sequences.

## Data Availability

The DNA sequence data generated during the current study are available in the European Nucleotide Archive (accession number: PRJEB39675). Additional datasets supporting this paper are available in the Supplementary Information.

## Abbreviations

aDNA: Ancient DNA
ADORA3: Adenosine A3 receptor
ADRAB2: Alpha 2B adrenergic receptor
ADRA2B: Alpha 2B adrenoceptor
APOB: Apolipoprotein B
APP: Amyloid beta precursor protein
BDNF: Brain-derived neurotrophic factor
BIC: Bayesian Information Criterion
BMI1: Polycomb complex protein BMI-1
BRCA1: Breast Cancer 1
BP: Years before present
bp: Base pairs
CREM: cAMP responsive element modulator
cyt *b*: Cytochrome *b*
HPD: Highest posterior density
Ma: Million years ago
MY: Million years
PCA: Principal component analysis
PC1: Principal component axis 1
PLCB4: Phospholipase C Beta 4
RAG1: Recombination activating gene 1
12S: 12S ribosomal RNA
