# Supplementary material for: Rapid size change associated with intra-island evolutionary radiation in extinct Caribbean “island-shrews”
Source: BMC Evol Biol. 2020 Aug 18;20:106. doi: 10.1186/s12862-020-01668-7 (PMC7437022; doi:10.1186/s12862-020-01668-7)
Supplement: Supplementary file 1 — Additional file 1: Figure S1. (a) Distribution of read lengths in paired-reads dataset. (b) Level of coverage across reference mitochondrial genome in mapped-reads dataset. Figure S2. Maximum likelihood phylogeny generated using RAxML, including Nesophontes paramicrus, N. zamicrus, and extant eulipotyphlans with available whole mitochondrial genome data. Node values represent bootstrap support (100 replicates). Table S1. Morphometric measurements taken on Hispaniolan Nesophontes mandibles. Table S2. Measurement and PCA data for Hispaniolan Nesophontes mandibles. Table S3. Genes used in phylogenetic analysis, including GenBank accession numbers. Asterisk indicates chimeric taxon made up of multiple species. Table S4. Evolutionary models chosen for each gene in the alignment using PartitionFinder. Table S5. Fossil constraints and priors used in divergence date analysis. Table S6. Eulipotyphlan species used in pairwise genetic distance analysis. Table S7. Pairwise distances for eulipotyphlan sister species pairs in the mitochondrial cyt b gene, showing number of base differences per site between sequences. Table S8. Pairwise distances for eulipotyphlan sister species pairs in the mitochondrial 12S gene, showing number of base differences per site between sequences. Table S9. Pairwise distances for eulipotyphlan sister species pairs in the CREM (cAMP responsive element modulator) protein-coding nuclear gene, showing number of base differences per site between sequences. Table S10. Pairwise distances for eulipotyphlan sister species pairs in the BRCA1 (Breast Cancer 1) protein-coding nuclear gene, showing number of base differences per site between sequences. Table S11. Pairwise distances for eulipotyphlan sister species pairs in the RAG1 (recombination activating gene 1) protein-coding nuclear gene, showing number of base differences per site between sequences. Table S12. Pairwise distances for eulipotyphlan sister species pairs in the BDNF (brain derived neurotrophic f [file 12862_2020_1668_MOESM1_ESM.zip › Woods_et_al_SI_figs+tables_revised.pdf]

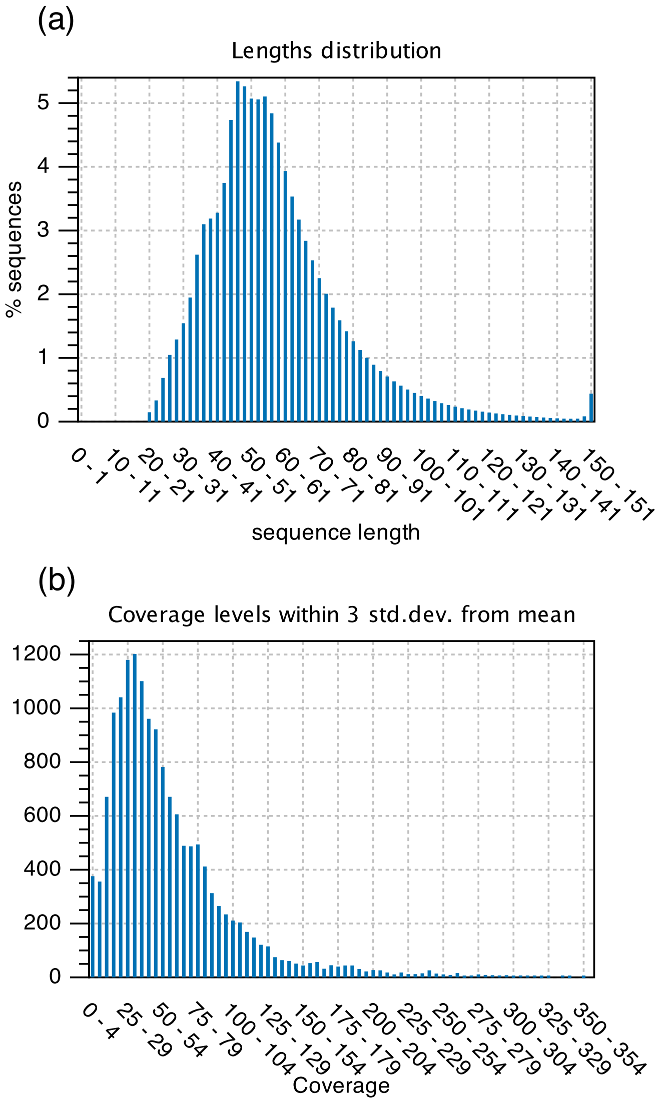

Figure S1. (a) Distribution of read lengths in paired-reads dataset. **(b)** Level of coverage across reference mitochondrial genome in mapped-reads dataset.

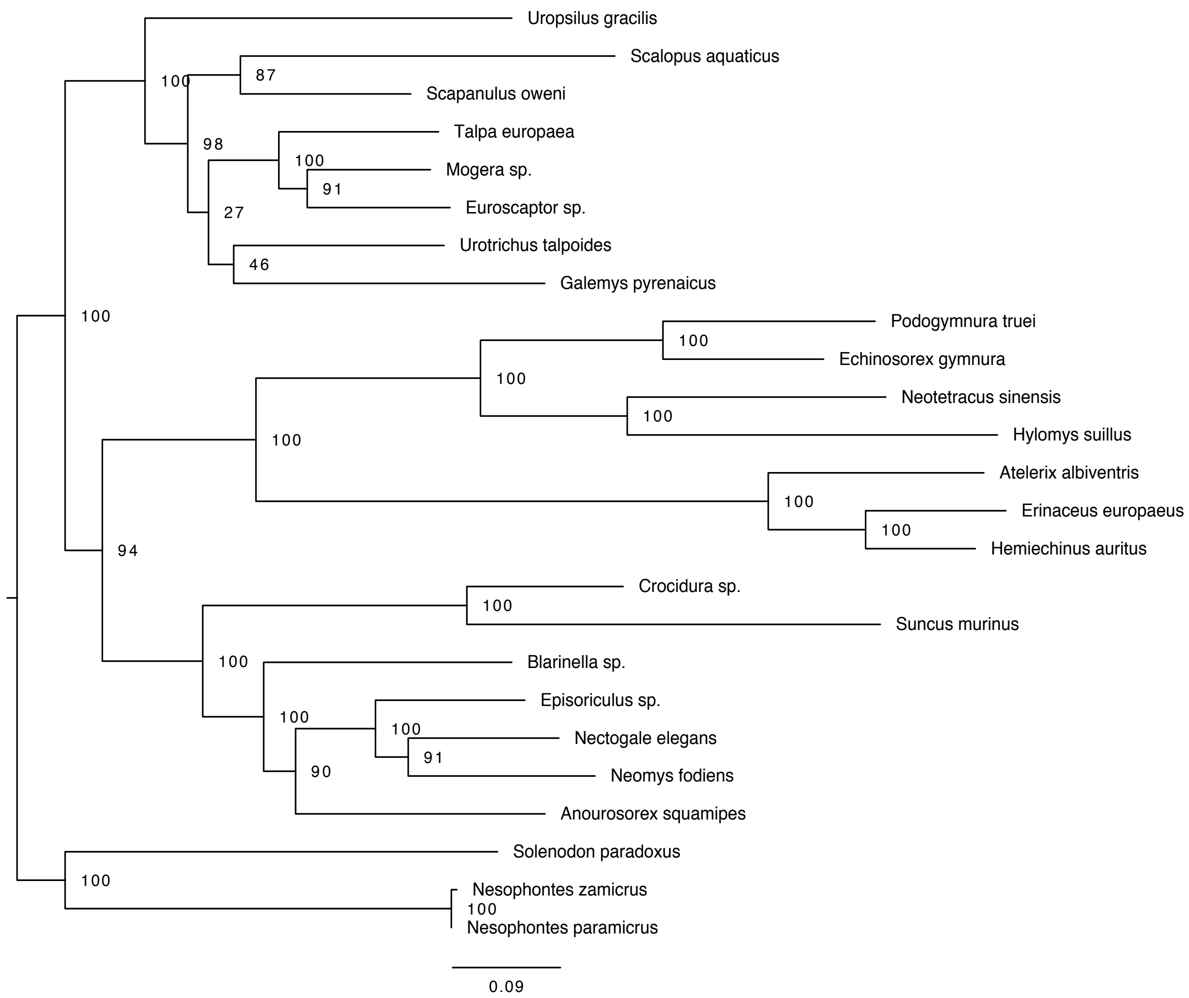

Figure S2. Maximum likelihood phylogeny generated using RAxML, including *Nesophontes paramicrus*, *N. zamicrus*, and extant eulipotyphlans with available whole mitochondrial genome data. Node values represent bootstrap support (100 replicates).

Table S1. Morphometric measurements taken on Hispaniolan *Nesophontes* mandibles.

Character	Description
1	Alveolar length of canine – m3
2	Alveolar length of m1 – m3
3	Length of canine – posterior margin of angular margin
4	Length of canine – posterior margin of condyle
5	Length of canine – dorsal margin of coronoid
6	Length of m3 – posterior margin of angular margin
7	Length of m3 – posterior margin of condyle
8	Length of m3 – dorsal margin of coronoid
9	Buccal width between m2 and m3
10	Width of condyle
11	Length of dorsal margin of coronoid – ventral margin of condyle
12	Length of dorsal margin of coronoid – ventral margin of angular margin
13	Length of dorsal margin of condyle – ventral margin of angular margin

Table S3. Genes used in phylogenetic analysis, including GenBank accession numbers. Asterisk indicates chimeric taxon made up of multiple species.

Family	Genus	Species	mt genome	APP	BMI1	CREM	PLCB4	ADORA3	APOB	ADRA2B	ADRB2
Nesophontidae	*Nesophontes*	*zamicrus*		X		X			X	X	X
Solenodontidae	*Solenodon*	*paradoxus*	X	AY530068	AY530071	AY451973,<br>AY530072	AY530074	AY530066	JN414026	AY530081	AY530067
Erinaceidae	*Erinaceus*	*europaeus*	NC.002080	XM.007519783<br>AY011319	XM.007526364	XM.007523995	XM.007539576	XM.007526934	JN414024	XM.007529903	XM.007516378
	Hemiechinus	*auritus*	NC.005033								
	Atelerix	*albiventris*	X					X	X	X	X
	Echinosorex	*gymnura*	NC.002808		AY986735	AY451978	AY451986				
	Hylomys	*suillus*	NC.010298				AY451987		DQ630204	AJ505819	
	Neotetracus	*sinensis*	NC.019626								
	Podogymnura	*truei*	AF434823,<br>AF434829,<br>AF298578-9	JN633548	AY986742	AY451980	JN633106	JN633426	JN414025	JN413881	JN633682
Soricidae	*Crocidura*	*russula/<br>*fuliginosa/<br>*gueldenstaedtii*	NC.006893		AY986736*<br>*gueldenstaedtii*	AY451977*<br>*gueldenstaedtii*	AY451985*<br>*gueldenstaedtii*		GU981117*<br>*fuliginosa*	AY121766	
	Suncus	*murinus/*remyi*	AB175074<br>AB032842<br>EF507245<br>AB032845<br>AB033720<br>F444469		AY986741	AY451981	AY451992		DQ630181		
			FJ814528<br>J813943								
			FJ813963<br>AB011390								
	Episoriculus	*fumidus*	NC.003040						DQ630193		
	Sorex	*araneus/<br>*unguiculatus*	NC.005435*	XM.004621155	AY011509	AY011631	AY011754	AY011200	JN414027	XM.004609236	XM.004620597
	Nectogale	*elegans*	NC.023351						GU981129		
	Blarinella	sp.	NC.023950						DQ630187		
	Anourosorex	*squamipes*	NC.024563								
	Neomys	*fodiens*	NC.025559						DQ630177		
Talpidae	*Mogera*	*wogura*	NC.005035	HG738058	HG738000	HG738020	HG738041	HG737959		AB638542	
	Talpa	*europaea/<br>*altaica*	NC.002391	HG738065*	HG738009	AY011629*	AY011752*	HG737968	JN414028*	JN413891*	AY011259*
	Euroscaptor	*mizura/*	AB106233	HG738051	HG737991	HG738012	HG738032	HG737949	DQ630168		

		**parvidens*	DQ630336, AB076828 DQ630413								
	Scalopus	*aquaticus*	AF069539 AY170059 AB076809							AF496636	
	Scapanulus	*oweni*	NC.025777								
	Condylura	*cristata*	X	XM.004675316	AY011508	AY011630	AY011753	AY011199	XM.004686059	XM.004686125	XM.004686868
	Galemys	*pyrenaicus*	NC.008156	AY833412		AY833411	AY833416	AY833418		AY121767	AY833413
	Urotrichus	*talpoides*	AB099483								
	Uropsilus	*gracilis*	NC.018598		KF778096	KF778144	KF778255	KF777913	DQ630199	AY121768	
Outgroups:											
Manidae	*Manis*	*pentadactyla*	NC.016008	AY011374	EF104992	AY011684	AY011807	AY011251	JN413999	JN413856	AY011311
Felidae	*Felis*	*catus*	NC.001700	AY011369	AY011553	AY011679	AY011802	AY011246	XM.003984468	XM.006930331	AY011306
Canidae	*Canis*	*lupus*	KF857179	GU167668	NM.001287063	GU167419	AY011805	NM.001003178	XM.005630596	XM.005642442	NM.001003234
Delphinidae	*Tursiops*	*truncatus*	NC.012059	AY011360	JQ002729	AY011670	AY011793	AY011237	JN413972	XM.004311473	AY011297
Bovidae	*Bos*	*taurus*	GU947019	NM.001076796		XM.005214245	NM.001166510	NM.001104611	XM.002691511	NM.001206628	NM.174231
Suidae	*Sus*	*scrofa*	NC.012095	AY011364	NM.001285971	AY011674	AY011797	AY011241	XM.001501679	NM.001037148	AY011301
Equidae	*Equus*	*caballus*	NC.001640	AY011366	XM.005606891	AY011676	AY011799	AY011243	JN414029	NM.001164012	AY011303
Vespertilionidae	*Myotis*	*davidii*	KF111724	XM.006758757	XM.006759928	XM.006774616	XM.006758082		XM.006767541	XM.006761143	XM.006766218
Pteropodidae	*Pteropus*	*alecto/ *giganteus*	NC.023122	XM.006908243	XM.006907001	XM.006911376	XM.006921597	XM.006919638	XM.006910423	XM.006909438	XM.006906373
Elephantidae	*Loxodonta*	*africana*	NC.000934	AY011326	AY011513	AY011635	AY011758	AY011204	JN413956	JN413853	AY011264

Table S4. Evolutionary models chosen for each gene in the alignment using Partitionfinder.

Gene partition	Model
**Mitochondrial genome**	
rrnS, trnC, trnF, trnL2, trnM, trnN, trnQ, trnR, trnS2, trnY	GTR + G
rrnL, trnD, trnH, trnI, trnK, trnL1, trnT, trnV, trnW	GTR + G
atp6, cob, nad1, nad3, nad4, trnA	GTR + G + I
atp8, nad2, nad4l, nad5, trnP	GTR + G
cox1, cox2, cox3, trnG	GTR + G + I
**Nuclear genes**	
ADORA3, ATP7A, GHR, VWF, BMI1, EDG1	GTR + G
CREM, APP, BRCA1, TTN	K80 + G
ADRB2, BDNF, RAG1, RAG2, BCHE	GTR + I

Table S5. Fossil constraints and priors used in divergence date analysis.

Root	Node	Fossil and deposit locality	Age (My)	Prior distribution
1	Laurasiatheria	Minimum: Earliest carnivoramorphan (*Protictis* from Fort Union/ Polecat Bench Formation)	Basal Torrejonian (To1) – Barremian	logNormalPrior mean="16.02" stdev="1.0" offset="62.5"
		Soft maximum: offset="62.5" Liaoning fossils of *Eomaia* and *Sinodelphys* (Theria)	62.5 – 131.5	
2	Laurasiatheria minus Eulipotyphla	*As above*	*As above*	*As above*
3	Ferungulata	*As above*	*As above*	*As above*
4	Erinaceidae/Soricidae	Minimum: Oldest erinaceomorph (*Adunator* from Torrejonian)	Early Danian – Barremian	logNormalPrior mean="16.25" stdev="1.0" offset="61.5"
		Soft maximum: *As above*	61.5 – 131.5	
5	Carnivora	Minimum: *Hesperocyon* from earliest Duchesnean	Earliest Duchesnean – Early Paleocene, 39.68 – 65.8	logNormalPrior mean="6.067" stdev="1.0" offset="39.68"
		Soft maximum: Oldest stem carnivorans in Torrejonian		
6	Cetartiodactyla	Minimum: *Himalayacetus* from base of Subathu Formation	Early Eocene – Early Paleocene, 52.4 – 65.8	logNormalPrior mean="3.111" stdev="1.0" Soft maximum: offset="52.4"
		Soft maximum: absence of crown Cetartiodactyla in Paleocene		
7	Chiroptera	Minimum: *Icaronycteris*	48.6 – 65.8	logNormalPrior mean="3.995" stdev="1.0" offset="48.6"
		Soft maximum: as for other members of Laurasiatheria		

Table S6. Eulipotyphlan species used in pairwise genetic distance analysis.

Species	Genetic distance ID	Species	Genetic distance ID
Mogera imaizumii	M1	*Neomys anomalus*	Neo1
Mogera wogura	M2	*Neomys teres*	Neo2
Mogera insularis	M3	*Neomys fodiens*	Neo3
Mogera tokudae	M4	*Suncus etruscus*	Su1
Euroscaptor klossi	Eu1	*Suncus murinus*	Su2
Euroscaptor malayana	Eu2	*Suncus montanus*	Su3
Euroscaptor longirostris	Eu3	*Suncus fellowesgordoni*	Su4
Euroscaptor mizura	Eu4	*Suncus varilla*	Su5
Euroscaptor parvidens	Eu5	*Suncus remyi*	Su6
Euroscaptor subanura	Eu6	*Suncus dayi*	Su7
Talpa stankovici	T1	*Suncus stoliczkanus*	Su8
Talpa europaea	T2	*Nesophontes paramicrus*	Nes1
Talpa altaica	T3	*Nesophontes zamicrus*	Nes2
Talpa caucasica	T4	*Atelerix albiventris*	A1
Talpa davidiana	T5	*Atelerix algirus*	A2
Uropsilus gracilis	U1	*Atelerix frontalis*	A3
Uropsilus nivatus	U2	*Erinaceus concolor*	Er1
Uropsilus soricipes	U3	*Erinaceus amurensis*	Er2
Uropsilus atronates	U4	*Erinaceus europaeus*	Er3
Uropsilus investigator	U5	*Erinaceus roumanicus*	Er4
Uropsilus aequodonenia	U6	*Anourosorex squamipes*	An1
Episoriculus caudatus	Ep1	*Anourosorex yamashinai*	An2
Episoriculus leucops	Ep2	*Blarinella griselda*	B1
Episoriculus macrurus	Ep3	*Blarinella quadraticauda*	B2
Episoriculus fumidus	Ep4	*Blarinella wardi*	B3

Table S7. Pairwise distances for eulipotyphlan sister species pairs in the mitochondrial cyt *b* gene, showing number of base differences per site between sequences. Analysis involved 29 nucleotide sequences. All ambiguous positions were removed for each sequence pair. The final dataset contained 1009 positions. Evolutionary analyses conducted in MEGA7.

	An1
An1	-
An2	0.061

	B1	B2
B1	-	
B2	0.048	-
B3	0.117	0.119

	Ep1	Ep2
Ep1	-	
Ep2	0.133	-
Ep3	0.156	0.156

	Neo1
Neo1	-
Neo3	0.146

	Su2
Su2	-
Su8	0.086

	Eu1	Eu2
Eu1	-	
Eu2	0.084	-
Eu4	0.134	0.125

	M1	M2	M4
M1	-		
M2	0.073	-	
M4	0.098	0.096	-
M3	0.096	0.111	0.111

	T3
T3	-
T2	0.137

	Nes1
Nes1	-
Nes2	0.022

	U1	U2	U6	U4
U1	-			
U2	0.025	-		
U6	0.102	0.103	-	
U4	0.138	0.132	0.142	-
U3	0.126	0.124	0.121	0.096

	A1
A1	-
A3	0.125

	Er3	Er4	Er2
Er3	-		
Er4	0.003	-	
Er2	0.109	0.110	-
Er1	0.128	0.129	0.131

Table S8. Pairwise distances for eulipotyphlan sister species pairs in the mitochondrial 12S gene, showing number of base differences per site between sequences. Analysis involved 29 nucleotide sequences. All ambiguous positions were removed for each sequence pair. The final dataset contained 1009 positions. Evolutionary analyses conducted in MEGA7.

	M1	M2	M3	
M1	-			
M2	0.025	-		
M3	0.032	0.034	-	
M4	0.027	0.034	0.029	
	Eu1	Eu2	Eu3	
Eu1	-			
Eu2	0.022	-		
Eu3	0.027	0.022	-	
Eu4	0.059	0.053	0.055	
	T1			
T1	-			
T2	0.095			
	U1	U2	U3	U4
U1	-			
U2	0.000	-		
U3	0.033	0.033	-	
U4	0.039	0.039	0.043	-
U5	0.032	0.032	0.037	0.042
	Ep1	Ep2	Ep4	
Ep1	-			
Ep2	0.039	-		
Ep3	0.063	0.057	-	
Ep4	0.080	0.072	0.071	
	Neo1	Neo2		
Neo1	-			
Neo2	0.035	-		
Neo3	0.053	0.045		
	Su1			
Su1	-			
Su2	0.071			
	Nes1			
Nes1	-			
Nes2	0.053			
	A1			
A1	-			
A2	0.023			

Table S9. Pairwise distances for eulipotyphlan sister species pairs in the CREM (cAMP responsive element modulator) protein-coding nuclear gene, showing number of base differences per site between sequences. Analysis involved 29 nucleotide sequences. All ambiguous positions were removed for each sequence pair. The final dataset contained 1009 positions. Evolutionary analyses conducted in MEGA7.

	T2		
T2	-		
T3	0.000		
	M2	M1	
M2	-		
M1	0.003	-	
M3	0.000	0.003	
	Eu2	Eu3	Eu5
Eu2	-		
Eu3	0.000	-	
Eu5	0.003	0.003	-
Eu4	0.005	0.005	0.003
	Nes1		
Nes1	-		
Nes2	0.022		

Table S10. Pairwise distances for eulipotyphlan sister species pairs in the BRCA1 (Breast Cancer 1) protein-coding nuclear gene, showing number of base differences per site between sequences. Analysis involved 29 nucleotide sequences. All ambiguous positions were removed for each sequence pair. The final dataset contained 1009 positions. Evolutionary analyses conducted in MEGA7.

	T2
T2	-
T4	0.004

	M2
M2	-
M3	0.013

	A3	A1
A3	-	
A1	0.002	-
A2	0.008	0.008

	Er3	Er2
Er3	-	
Er2	0.005	-
Er4	0.005	0.005

	Su5	Su6	Su2	Su7
Su5	-			
Su6	0.038	-		
Su2	0.072	0.088	-	
Su7	0.074	0.079	0.031	-
Su1	0.075	0.082	0.056	0.058

	Nes2
Nes2	-
Nes1	0.004

Table S11. Pairwise distances for eulipotyphlan sister species pairs in the RAG1 (recombination activating gene 1) protein-coding nuclear gene, showing number of base differences per site between sequences. Analysis involved 29 nucleotide sequences. All ambiguous positions were removed for each sequence pair. The final dataset contained 1009 positions. Evolutionary analyses conducted in MEGA7.

	T4	T3	T2
T4	-		
T3	0.018	-	
T2	0.022	0.021	-
T5	0.022	0.022	0.024
	M2	M1	M4
M2	-		
M1	0.003	-	
M4	0.008	0.005	-
M3	0.016	0.015	0.016
	Eu4	Eu2	
Eu4	-		
Eu2	0.012	-	
Eu6	0.019	0.019	
	U1		
U1	-		
U4	0.007		
	Su2	Su3	Su4
Su2	-		
Su3	0.005	-	
Su4	0.035	0.036	-
Su1	0.032	0.032	0.016
	Nes2		
Nes2	-		
Nes1	0.001		
	Er3	Er2	
Er3	-		
Er2	0.003	-	
Er4	0.004	0.001	
	A2	A1	
A2	-		
A1	0.002	-	
A3	0.006	0.007	

Table S12. Pairwise distances for eulipotyphlan sister species pairs in the BDNF (brain derived neurotrophic factor) protein-coding nuclear gene, showing number of base differences per site between sequences. Analysis involved 29 nucleotide sequences. All ambiguous positions were removed for each sequence pair. The final dataset contained 1009 positions. Evolutionary analyses conducted in MEGA7.

	Su3	Su4	Su1
Su3	-		
Su4	0.000	-	
Su1	0.000	0.000	-
Su2	0.000	0.000	0.000
	T2	T3	
T2	-		
T3	0.006		
	Eu2	Eu3	Eu4
Eu2	-		
Eu3	0.006	-	
Eu5	0.010	0.008	-
Eu4	0.015	0.014	0.010
	M2	M1	
M2	-		
M1	0.000	-	
M3	0.006	0.006	
	Nes2		
Nes2	-		
Nes1	0.006		
	Er1		
Er1	-		
Er2	0.002		
